# O8-4 The effect of The Daily Mile on primary school children's aerobic fitness levels after 12 weeks: a controlled trial

**DOI:** 10.1093/eurpub/ckac094.060

**Published:** 2022-08-29

**Authors:** Maxine de Jonge, Jorien Slot-Heijs, Rick Prins, Amika Singh

**Affiliations:** Mulier Instituut, Utrecht, The Netherlands

**Keywords:** The Daily Mile, school setting, aerobic fitness

## Abstract

**Background:**

The Daily Mile is a school-based physical activity intervention that aims to improve children's aerobic fitness levels. It encompasses a 15-minute run for the whole class on or around the school grounds at least three times per week. The Daily Mile is an easy and accessible intervention, but the downside is a low threshold to stop or skip a session. Therefore, we aimed to determine 1) the effects of performing The Daily Mile for 12 weeks on the aerobic fitness levels of Dutch primary school children and 2) if additional personal support for teachers impacted the effectiveness of The Daily Mile.

**Methods:**

We conducted a controlled trial in grades 5 through 8 of nine primary schools across the Netherlands. Schools were allocated to control, intervention (12 weeks The Daily Mile) or intervention-plus (12 weeks The Daily Mile and additional support) group. Children completed the shuttle-run test (SRT) at baseline and follow-up, 12 weeks apart. We analyzed the data using multi-level linear regression models clustered within individuals and by classes and schools. All models were adjusted for sex and age.

**Results:**

We collected complete data sets for 536 children (mean age 10.0 years). The participation rate of classes in the intervention group was 87.8% and in the intervention-plus group 89.7%. After correcting for age and sex, the adjusted model showed a significant intervention effect on SRT-score after 12 weeks for both the intervention group (1.1 stages; 95% CI 0.75, 1.47) and the intervention-plus group (0.6 stages; 95% CI 0.32, 0.89), when compared to the control group.

**Conclusion:**

Performing The Daily Mile at least three times per week for a 12 week period can be effective in increasing the aerobic fitness levels of primary school children. Additional personal support for teachers did not improve the effectiveness of the intervention on aerobic fitness within this time frame. This may be due to the high implementation rates in both the intervention group and the intervention-plus group. Possibly additional personal support might become beneficial for maintenance on the longer term.

